# Dietary Creatine and Hydration Biomarkers in the General Population: NHANES 1999–2023

**DOI:** 10.1002/fsn3.70524

**Published:** 2025-06-27

**Authors:** Sergej M. Ostojic, Erik Grasaas

**Affiliations:** ^1^ Faculty of Health Sciences University of Pécs Pécs Hungary; ^2^ Department of Nutrition and Public Health University of Agder Kristiansand Norway; ^3^ Teacher Education Unit University of Agder Kristiansand Norway

**Keywords:** creatine, diet, hydration, NHANES, osmolality, total body water

## Abstract

Creatine is widely recognized for its performance‐enhancing and neuroprotective properties; however, its impact on hydration status at the population level remains poorly understood. This study investigated the relationship between dietary creatine intake and hydration biomarkers in a nationally representative sample derived from the National Health and Nutrition Examination Survey (NHANES). Data from the biannual 1999 and 2023 NHANES cycles were harmonized and analyzed, including dietary recall data and hydration biomarkers such as plasma osmolality, urine specific gravity (USG), total body water (TBW), intracellular fluid (ICF), and extracellular fluid (ECF) volumes. Valid data on dietary creatine intake were obtained from 98,681 participants, with serum osmolality measured in 69,144 individuals, fluid volumes assessed in 12,369, and USG evaluated in 6274 respondents. Total daily dietary creatine intake was expressed in milligrams per kilogram of body mass and categorized into quantiles. Associations were evaluated using multivariate regression models, adjusted for potential confounders, including gender, age, daily intake of caffeine, alcohol, sodium, water, protein, and total caloric intake. Both the lowest and highest creatine intake quantiles were significantly associated with altered plasma osmolality levels and an increased prevalence of hypoosmolality (*p* < 0.01), relative to moderate creatine intake (4.27–10.19 mg/kg). Higher creatine intake was also associated with reduced TBW, ICF, and ECF volumes (*p* < 0.01). Notably, a significantly lower risk of hypoosmolality was observed in the 50th percentile (OR = 0.861; *p* = 0.035) and the 75th percentile (OR = 0.832; *p* = 0.010) of creatine intake. Our findings challenge the prevailing notion that dietary creatine universally enhances hydration. While moderate intake from regular foods appears to have a neutral effect, higher intake levels may disrupt systemic fluid balance. Further research is warranted to explore the underlying physiological mechanisms and evaluate the long‐term effects of creatine intake on hydration homeostasis.

## Graphical abstract

Our findings indicate a complex relationship between dietary creatine intake and systemic hydration, with both low and high intake levels are associated with changes in plasma osmolality and hydration markers, whereas moderate intake shows minimal effects. Notably, higher creatine intake is linked to reductions in total body water (TBW), intracellular fluid (ICF), and extracellular fluid (ECF) volumes. These results challenge the traditional view that creatine consistently improves hydration and underscore the need for further investigation into its role in fluid balance.

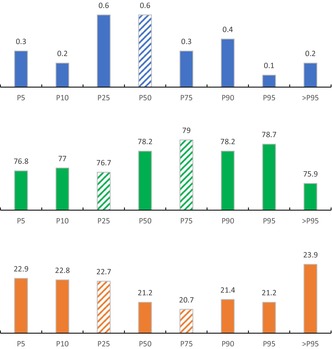

## 1 Introduction

Creatine is a naturally occurring nonproteinogenic amino acid derivative that is regarded as a semiessential nutrient in human nutrition (Ostojic and Forbes 2022). It plays a pivotal role in cellular bioenergetics, primarily through its involvement in the phosphocreatine system, which enables the rapid regeneration of adenosine triphosphate (ATP)—a crucial energy molecule essential for cellular function in living organisms (Wallimann et al. 2011). The use of creatine in human nutrition dates back to the 1920s, when it was first proposed as a “reserve food material” that could be stored in the body following dietary intake (Chanutin and Guy 1926). Scientific interest in creatine expanded significantly in the 1990s, initially within the field of sports nutrition, where it was investigated for its ergogenic benefits. Subsequent research broadened its scope to include clinical applications across various medical conditions (for a detailed review, see Kreider and Stout 2021). Beyond its well‐established role in energy metabolism, creatine has been explored for its potential impact on body hydration, given its function as an osmotically active compound. Several studies have reported that creatine supplementation increases total body water (TBW) (Francaux and Poortmans 1999; Powers et al. 2003; Brilla et al. 2003; Buck et al. 2023), primarily due to an expansion in intracellular fluid (ICF) volume, facilitated by sodium‐dependent creatine transport mechanisms. Conversely, other investigations have found no significant changes in TBW following creatine intake (Kreider et al. 1998; Rawson et al. 2011) or even potential dehydration effects (Bailes et al. 2002), leading to ongoing debate regarding its effects on hydration status. Notably, both supporting and contradicting studies were typically small‐scale, of short duration, utilized widely varying amounts of creatine, and primarily conducted in exercise settings, thereby limiting their generalizability to broader populations. To date, no large‐scale epidemiological studies have systematically examined the potential relationship between dietary creatine intake and hydration status at the population level, while accounting for multiple biomarkers of hydration and critical covariates influencing water homeostasis. Therefore, the primary aim of this cross‐sectional epidemiological study was to investigate the association between dietary creatine consumption and various hydration indicators in a nationally representative sample of US individuals.

## 2 Methods

### 2.1 Study Population

In this cross‐sectional, population‐based study, we analyzed data from the National Health and Nutrition Examination Survey (NHANES), a continuously conducted program of biannual surveys designed to assess the health and nutritional status of a representative sample of individuals from the US civilian, noninstitutionalized population. NHANES collects comprehensive data, including demographic characteristics, lifestyle routines and health indicators, obtained through physical examinations, laboratory tests, and structured interviews on dietary habits and health‐related conditions. The detailed methodology of NHANES has been extensively described elsewhere (US Department of Health and Human Services, 2024). For this analysis, we compiled and harmonized NHANES data from March 1999 to August 2023, selecting participants who provided a single in‐person 24‐h dietary recall as well as at least one hydration status indicator, sourced from the Examination Data and/or Laboratory Data databases (see below). Ethical approval for the NHANES study was granted by the National Center for Health Statistics Ethics Review Board under the following protocols: #98‐12, #2005‐06, Continuation of Protocol #2005‐06, #2011‐17, Continuation of Protocol #2011‐17, #2018‐01, Continuation of Protocol #2018‐01, and #2021‐05. Informed consent was obtained from all participants prior to their inclusion in the study. The research was conducted ethically following the World Medical Association Declaration of Helsinki.

### 2.2 Study Outcomes, Exposures and Covariates

The study outcomes (dependent variables) included fluid volume measurements obtained through bioelectrical impedance analysis (BIA), specifically TBW, ICF, and extracellular fluid (ECF). Additionally, clinical chemistry biomarkers of hydration were assessed, including plasma osmolality and urine specific gravity (USG). Plasma osmolality was analyzed both as a continuous variable and as a categorical variable, with participants classified into three hydration status groups: hyperosmolality (> 295 mOsm/kg), isoosmolality (275–295 mOsm/kg), and hypoosmolality (< 275 mOsm/kg) (Najem et al. 2025). The primary exposure (independent) variables included total daily dietary creatine intake, expressed in milligrams per kilogram of body mass, as well as categorical classification of creatine intake levels across percentile quantiles. The data were stratified into eight quantile groups (P5, P10, P25, P50, P75, P90, P95, and > P95) to enhance the detection of potential nonlinear or threshold effects that may not be apparent in continuous analyses alone. This stratification enables a more granular visualization of the dose–response relationship and supports clinical interpretation by delineating specific exposure intervals associated with significant changes in the outcome. Moreover, the quantile‐based analysis serves as a complementary approach to the continuous models, reinforcing the robustness of the observed associations. Potential confounders included gender, age at screening, and daily intake of caffeine, alcohol, sodium, water, protein, and total calories.

### 2.3 Data Sources and Measurements

The BIA examination was conducted on eligible survey participants aged 8–49 years from NHANES 1999–2004 as part of the Body Composition component in the Mobile Examination Center (MEC). Exclusion criteria included pregnancy, limb amputations (excluding fingers or toes), the presence of artificial joints or implanted metal objects (e.g., pacemakers, automatic defibrillators, coronary stents), and a body weight exceeding 136 kg. Participants were required to fast for at least 6 h before the assessment. BIA measurements were performed using a bioimpedance spectrum analyzer (HYDRA ECF/ICF Model 4200, Xitron Technologies Inc., San Diego, CA), a multifrequency device that utilizes a 12‐bit digital signal processing technique to measure impedance at 50 frequencies logarithmically spaced from 5 kHz to 1 MHz. Trained health technicians conducted the BIA assessments, collecting raw frequency data, including resistance and reactance measurements; the procedure took approximately 15 s per participant. The equations used to estimate fluid volumes have been described in previous studies (Patel et al. 1994; Plank et al. 1998), and a detailed explanation of the BIA examination protocol and data processing can be found in other sources (NHANES 2000). Serum osmolality was calculated in all participants aged 12 years and older from NHANES 1999–2020 using the following equation: serum osmolality = 1.86 × [Na^+^ (mmol/L)] + glucose (mg/dL)/18 + blood urea nitrogen (BUN; mg/dL)/2.8 + 9 (Dorwart and Chalmers 1975). Serum sodium levels were measured using the DxC800 system, which employs an indirect (diluted) ion‐selective electrode methodology (Beckman Coulter, Brea, CA). Glucose concentration was determined using the oxygen rate method with a Beckman Oxygen Electrode (glucose oxidase method). BUN concentration was quantified in serum using the enzymatic conductivity rate method. Detailed laboratory procedures and quality control measures for serum sodium, glucose, and BUN measurements are available elsewhere (CDC/National Center for Health Statistics 2022). USG was measured in all NHANES 2007–2008 participants aged 6 years and older using a digital handheld refractometer (Atago PAL‐10S, Tokyo, Japan) with automatic temperature compensation. Daily creatine intake was assessed for all NHANES 1999–2023 participants using dietary data from individual in‐person 24‐h food recall interviews, with proxies providing responses for participants under 6 years of age. Intake calculations were based on the average creatine content of various food sources (e.g., 0.20 g/kg for milk‐based foods and 3.88 g/kg for meat‐based sources), as previously described (Baltic et al. 2024). Creatine intake from nutritional supplements or pharmacological sources was excluded from the analysis. Relevant confounding variables were obtained from the Demographics, Dietary, Examination and Laboratory Data databases.

### 2.4 Statistical Analyses

A comparative analysis of hydration biomarkers across creatine intake quantiles was conducted using one‐way analysis of variance (ANOVA), followed by Tukey's post hoc test to identify pairwise differences between groups. The prevalence of hyperosmolality, normal osmolality, and hypoosmolality across creatine intake quantiles was assessed using adjusted chi‐squared tests, with post hoc comparisons to determine specific group differences. To examine the association between dietary creatine intake and hydration biomarkers, both crude linear regression models and models adjusted for an a priori‐defined set of covariates were employed. Additionally, multinomial logistic regression analysis was performed to evaluate the relationship between hydration status categories and quintiles of dietary creatine intake. All statistical analyses were conducted using two‐tailed values, with statistical significance set at *p* ≤ 0.05. Statistical computations were performed using IBM SPSS Statistics for Mac (version 24.0; IBM Corp., Chicago, IL, USA).

## 3 Results

After excluding duplicate entries and incomplete data, a total of 98,681 respondents from the NHANES 1999–2023 cumulative rounds provided valid information on dietary creatine intake. Among them, serum osmolality was measured in 69,144 participants, fluid volumes were assessed in 12,369 individuals, and USG was measured in 6274 respondents. The demographic characteristics and relevant dietary and examination data of the study participants are depicted in Table 1.

**TABLE 1:** Characteristics of the study population.

Variables		*n*
Demographics		
Age (years), mean ± SD	32.2 ± 24.8	108,403
≤ 17.9 years	39.5	
18.0–64.9 years	46.1	
≥ 65.0 years	14.4	
Female, %	50.9	
Body mass index (kg/m^2^), mean ± SD	26.0 ± 7.8	91,381
Dietary data		
Total energy intake (kcal/day), mean ± SD	1988 ± 973	107,033
Protein intake (g/day), mean ± SD	73.0 ± 41.0	107,033
Water intake^a^ (kg/day), mean ± SD	2.3 ± 1.4	88,612
Sodium intake (g/day), mean ± SD	3.0 ± 1.7	107,033
Alcohol intake (g/day), mean ± SD	166.4 ± 573.9	98,312
Caffeine intake (g/day), mean ± SD	88.2 ± 164.0	107,033
Creatine intake (mg/kg/day), mean ± SD	15.0 ± 17.1	98,681
Hydration biomarkers		
Plasma osmolality (mOsm/kg), mean ± SD	278.4 ± 5.4	69,144
mOsm/kg, %	0.5	
275–295 mOsm/kg, %	77.8	
mOsm/kg, %	21.7	
Urine specific gravity, mean ± SD	1.018 ± 0.08	6274
Total body water (L), mean ± SD	34.2 ± 11.2	12,369
Extracellular fluid (L), mean ± SD	14.7 ± 4.2	12,369
Intracellular fluid (L), mean ± SD	19.6 ± 7.2	12,369

^a^ Value reflects moisture present in all foods, beverages, and water consumed as a beverage.

The mean daily creatine intake among all participants was 15.0 ± 17.1 mg per kg of body weight (95% confidence interval [CI]: 14.9–15.1 mg/kg; median 10.2 mg/kg). The highest recorded intake (544.3 mg/kg) was observed in a 15‐month‐old male participant. In addition, a total of 8266 respondents (8.4%) reported no creatine intake. The percentile quantiles of creatine intake and their corresponding mean ± SD values were depicted in Table 2.

**TABLE 2:** Quantiles of daily creatine intake in milligrams per kilogram of body mass.

	P5	P10	P25	P50	P75	P90	P95	> P95
Range	0.00–0.09	0.10–0.79	0.80–4.26	4.27–10.19	10.20–19.89	19.90–33.80	33.81–45.76	> 45.76
Mean	0.00	0.41	2.48	7.15	14.47	25.64	38.99	68.35
SD	0.01	0.21	1.03	1.69	2.74	3.89	3.41	27.41

A comparison of creatine intake across different quantiles revealed significant differences among intake categories for all measured biomarkers of hydration (*p* < 0.01) (Table 3). Specifically, plasma osmolality was significantly higher in the P50 percentile rank compared to both the lowest (P5) and highest (>P95) creatine intake groups. Additionally, USG was significantly lower in the P10 percentile rank compared to higher intake categories, suggesting a potential impact of low creatine intake on renal concentration ability. Notably, all measured fluid volume parameters, including TBW, ECF, and ICF, exhibited significantly lower values in higher percentile ranks of creatine intake.

**TABLE 3:** Hydration biomarkers across quantiles of creatine intake. Values are mean ± SD.

	P5	P10	P25	P50	P75	P90	P95	> P95	p*
Osmolality (mOsm/kg)	278.1	278.2	278.3	278.6^a b c^	278.4^a^	278.2^d e^	278.2	278.0^d^	< 0.01
	5.4	5.1	5.5	5.5	5.4	5.2	4.9	5.0	
Urine specific gravity	1.017	1.016	1.017	1.018^b^	1.018^b c^	1.018^b^	1.019^b^	1.018	< 0.01
	0.008	0.007	0.007	0.008	0.007	0.007	0.007	0.007	
Total body water (L)	35.0	36.2	34.1^b^	36.0	34.7^d^	33.5^b d e^	30.8^a b c d e f^	28.9^a b c d e f^	< 0.01
	10.5	9.6	10.8	11.2	11.6	11.5	11.1	11.3	
Extracellular fluid (L)	15.0	15.5	14.7^b^	15.3^c^	14.8^b d^	14.3^a b d e^	13.2^a b c d e f^	12.4^a b c d e f^	< 0.01
	4.0	3.5	4.0	4.2	4.3	4.2	4.1	4.2	
Intracellular fluid (L)	20.1	20.6	19.5	20.6^c^	19.9	19.2^b d^	17.6^a b c d e f^	16.4^a b c d e f^	< 0.01
	6.7	6.3	7.0	7.3	7.5	7.4	7.1	7.2	

*Note:* An asterisk (*) indicates statistical significance assessed by one‐way ANOVA across quantiles of creatine intake. Superscript letters denote significant differences at *P* ≤ 0.05 between individual quantiles, as follows: ^a^denotes a difference from P5; ^b^denotes a difference from P10; ^c^denotes a difference from P25; ^d^denotes a difference from P50; ^e^denotes a difference from P75; ^f^denotes a difference from P90.

**FIGURE 1:**
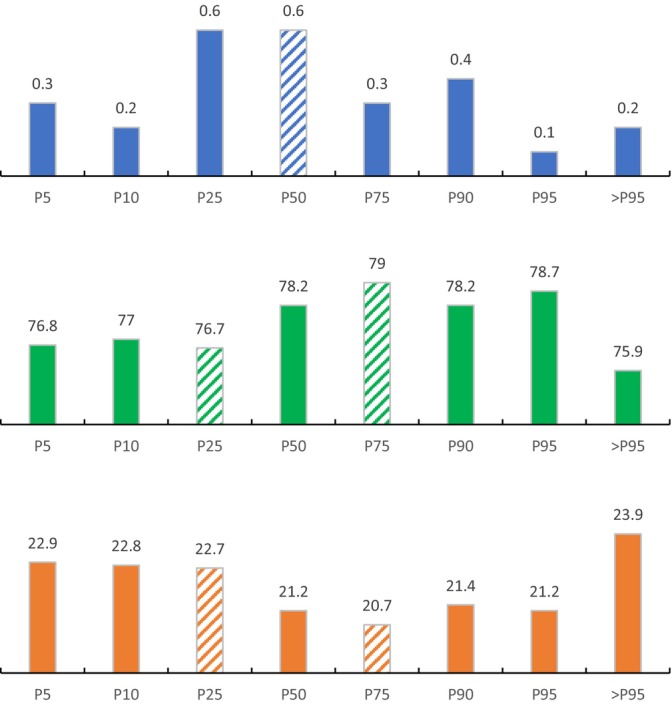
Prevalence of hyperosmolality (blue), normal osmolality (green) and hypoosmolality (orange) (%) across quantiles of creatine intake among NHANES 1999–2023 respondents. Quantiles that differ significantly (*p* < 0.05) are indicated by a distinct diagonal stripes pattern within the corresponding column.

In addition, the prevalence of hyperosmolality, normal osmolality, and hypoosmolality varied significantly across different quantiles of creatine intake (*p* < 0.01) (Figure 1); the lowest prevalence of hypoosmolality and hyperosmolality was observed at the 75th percentile (20.7%) and 95th percentile (0.1%), respectively.

A crude linear regression analysis indicated a strong but nonsignificant trend toward a negative association between dietary creatine intake and plasma osmolality (*B* = −0.003, *p* = 0.08). In contrast, significant associations were observed between dietary creatine intake and USG (*B* = 0.00003, *p* < 0.01), TBW (*B* = −0.107, *p* < 0.01), ECF (*B* = −0.046, *p* < 0.01), and ICF (*B* = −0.061, *p* < 0.01). For example, each 1 mg/kg increase in dietary creatine intake was associated with an expected increase of 0.00003 units in USG, and expected decreases of 0.107 L, 0.046 L, and 0.061 L in TBW, ECF, and ICF, respectively. These associations remained statistically significant after adjusting for potential confounders across all hydration biomarkers, including plasma osmolality (*B* = −0.004, *p* = 0.05), USG (*B* = −0.00003, *p* < 0.01), TBW (*B* = −0.227, *p* < 0.01), ECF (*B* = −0.089, *p* < 0.01), and ICF (*B* = −0.139, *p* < 0.01). Interestingly, the multinomial logistic regression analysis showed a significantly lower risk of hypoosmolality in the P50 (OR = 0.861; 95% CI: 0.750–0.989; *p* = 0.035) and P75 (OR = 0.832; 95% CI: 0.724–0.956; *p* = 0.010) creatine intake quantiles, compared to the reference category of normal osmolality.

## 4 Discussion

Our epidemiological study provides one of the first comprehensive insights into the relationship between dietary creatine intake and hydration biomarkers in a large, diverse population. The findings reveal significant variations in hydration status across different levels of creatine intake, as represented by percentile quantiles. Notably, both the lowest and highest creatine intake quantiles are associated with significantly different plasma osmolality levels and a higher prevalence of hypoosmolality (more diluted plasma) compared to mid‐range intake levels, suggesting a potential nonlinear relationship between creatine consumption and systemic hydration. Additionally, higher creatine intake (P75 and above) is linked to significantly lower fluid volume parameters compared to lower intake levels (P50 and below), with these associations remaining significant even after adjusting for relevant nutritional and demographic factors, highlighting a potential association between creatine intake and body fluid distribution. These results suggest that moderate daily creatine intake (4.27–10.19 mg/kg) may not substantially impact hydration biomarkers, whereas higher intakes could be associated with reduced body hydration. However, these findings should be interpreted with caution and require further investigation.

Creatine is frequently discussed in the context of body hydration due to its osmotically active properties and its capacity to enhance intracellular water retention. Exogenous creatine supplementation increases the intracellular concentrations of creatine and phosphocreatine within skeletal muscle (Hultman et al. 1996), thereby creating an osmotic gradient that facilitates water influx into myocytes, a phenomenon known as cell volumization. Multiple studies have demonstrated that creatine supplementation can acutely enhance cellular hydration (Francaux and Poortmans 1999; Bemben et al. 2001; Brilla et al. 2003; Buck et al. 2023). While some evidence suggests that creatine may also influence extracellular water retention (Moore et al. 2023), research remains inconclusive regarding whether creatine supplementation significantly alters the balance between ICF and ECF or simply increases TBW without disrupting hydration homeostasis (Powers et al. 2003). Conversely, several studies have reported no significant effects of creatine supplementation on hydration markers (Burke et al. 2003; Aedma et al. 2015), challenging the prevailing assumption that creatine induces water retention. Additionally, speculative reports have suggested that potential intramuscular fluid shifts could influence plasma volume and contribute to dehydration (Bailes et al. 2002); however, strong empirical evidence supporting this hypothesis is lacking. The conflicting or inconclusive findings have sustained ongoing debate regarding the relationship between creatine supplementation and hydration status (Lopez et al. 2009). Several factors may contribute to the variability in results across studies, including substantial differences in creatine dosage (up to 300 mg per kg body weight per day), varying supplementation durations (from single‐dose acute studies to medium‐term trials), differences in creatine formulations (creatine monohydrate vs. other forms), and limited diversity in study populations, which often consist primarily of athletes or young physically active individuals. Furthermore, key confounding variables—such as heat stress, dietary factors (particularly fluid intake), small sample sizes, and inconsistent methodologies for assessing body hydration—have frequently been overlooked.

To address these limitations, our cross‐sectional study expanded upon previous research by utilizing a large, diverse sample encompassing participants across a wide age range. We systematically analyzed dietary creatine exposure across a broad spectrum of intakes while assessing relevant circulatory and urinary biomarkers of hydration and body composition. The dietary creatine intake assessed in this study stands in contrast to creatine supplementation, as the estimated daily exposure from habitual diet (~ 15.0 mg/kg body weight) is substantially lower than that typically achieved through supplementation protocols (~ 60 mg/kg body weight per day). Additionally, we controlled for critical hydration‐related confounding variables from the diet, thereby providing a more comprehensive understanding of the potential links between creatine intake and hydration status. The stratification of creatine intake into eight percentile‐based quantiles revealed significant differences in hydration biomarkers across groups. Notably, plasma osmolality was significantly higher in the P50 percentile compared to both the lowest (P5) and highest (>P95) intake groups, suggesting that moderate creatine consumption (4.27–10.19 mg/kg) may be associated with improved body's electrolyte‐water balance. In contrast, individuals in the P10 percentile, representing the second‐lowest creatine intake group, exhibited significantly lower USG compared to those in higher intake quantiles (P50 and above). This pattern may reflect a diminished renal concentrating ability in response to low creatine availability, potentially affecting water retention and urinary excretion mechanisms. Further analyses indicated that higher creatine intake was associated with a reduction in total TBW, ECF, and ICF volumes, particularly in the upper intake percentiles. This finding suggests that creatine may influence body fluid distribution, with a trend indicating that excessive creatine intake from food sources could be linked to lower overall fluid volumes. These observations were further supported by multinomial logistic regression analysis, which demonstrated a significantly lower risk of hypoosmolality in the upper creatine intake quantiles. This suggests that higher creatine intake may reduce the likelihood of developing diluted plasma. However, excessive creatine intake may induce osmotic stress, potentially drawing disproportionate amounts of water into skeletal muscle and the gastrointestinal tract, as previously suggested (Bailes et al. 2002). This fluid redistribution could disrupt the balance between intracellular and extracellular compartments, potentially leading to transient bloating, electrolyte imbalances, and reductions in plasma volume. Conversely, moderate creatine intake appears to optimize intracellular hydration without the adverse effects associated with excessive dosing (Persky and Rawson 2007). This supports the notion that, within physiological limits, creatine intake may contribute to hydration homeostasis when consumed at appropriate levels, rather than at extreme intakes.

The average daily creatine intake in our study population was 15.0 mg/kg body weight, aligning with findings from previous epidemiological studies (Korovljev et al. 2021). Among the participants, 34,921 individuals (35.4%) consumed creatine at or above the population mean, while a substantial proportion (8.4%) reported no creatine intake. Notably, individuals with little to no creatine consumption exhibited slightly lower plasma osmolality and USG, suggesting a reduced availability of creatine as an osmotically active compound capable of contributing to hydration homeostasis. Several physiological mechanisms may account for the observed relationship between low creatine intake and alterations in hydration biomarkers. First, a reduced osmotic drive for intracellular water retention due to lower creatine availability may diminish ICF retention, leading to less efficient water distribution within muscle cells. Second, a lower osmotic load may result in increased excretion of free water, contributing to a more dilute urine profile. Third, creatine has been implicated in modulating sodium and electrolyte balance (Willott et al. 1999), and its absence may lead to greater sodium excretion and lower ECF retention. Fourth, creatine plays a role in muscle energy metabolism, and its reduced intake may be associated with lower muscle glycogen stores (van Loon et al. 2004), thereby decreasing intracellular water retention. Additional physiological or metabolic processes may contribute to the observed reductions in plasma osmolality and USG, and even a combination of multiple mechanisms; it is likely that several of these mechanisms interact, collectively influencing hydration status in individuals with minimal creatine intake. The relative contribution of each mechanism, as well as the magnitude of their isolated and combined effects on plasma osmolality and USG, remains unclear and warrants further investigation. Future research should aim to quantify these effects through controlled trials and large‐scale epidemiological studies, considering potential confounding factors such as fluid intake, dietary patterns, physical activity levels, and environmental conditions.

Our findings should be interpreted with caution when comparing them to results from creatine supplementation studies. In addition to previously discussed differences in study design (longitudinal vs. cross‐sectional), creatine exposure levels, and demographic diversity, the molecular composition of creatine derived from food sources versus dietary supplements may also differ, potentially influencing hydration regulation. While creatine from food sources is predominantly in its free form (C_4_H_9_N_3_O_2_), the majority of dietary supplements provide creatine as creatine monohydrate (C_4_H_9_N_3_O_2_·H_2_O), which consists of one creatine molecule bound to a single water molecule. This structural difference suggests that creatine monohydrate may contribute an additional water molecule to overall body water balance. Although this effect is likely minimal compared to the more substantial impact of creatine's osmotic properties, it may confer a slight advantage in promoting intracellular hydration compared to food‐derived creatine. Consequently, higher dietary intake of creatine from food sources may influence hydration status differently than supplemental creatine monohydrate. To elucidate these potential differences, further research employing biochemical assays (e.g., aquaporin expression studies and deuterium oxide dilution) and long‐term hydration studies is necessary to determine whether food‐derived creatine, creatine monohydrate, and other supplemental creatine forms vary in their effects on water retention, osmoregulation, and hydration‐related health outcomes.

The present study utilizes NHANES data to examine the relationship between dietary creatine intake and hydration status markers. While leveraging a large, nationally representative sample enhances generalizability, several limitations should be acknowledged. The cross‐sectional design precludes causal inference, limiting the ability to determine whether creatine intake influences hydration markers or vice versa. Additionally, the reliance on a single 24‐h dietary recall to assess creatine intake introduces potential measurement error and recall bias, particularly given the exclusion of creatine from supplements or pharmacological sources. The study also faces limitations in hydration assessment, as BIA was only available for a subset of participants, restricting sample size and generalizability across age groups. Furthermore, plasma osmolality was estimated using a formula rather than direct measurement in some cases, potentially affecting accuracy. The exclusion of individuals with certain medical conditions, as well as those exceeding weight thresholds, may limit external validity. Finally, although a potential nonlinear relationship between creatine intake and the outcomes was acknowledged, our analyses were primarily designed to capture general trends using both categorical and continuous representations; future studies employing advanced modeling approaches (e.g., spline regression or generalized additive models) are warranted to further explore potential nonlinear associations. Despite these constraints, the study provides valuable insights into the associations between dietary creatine intake and hydration markers, but future research should employ longitudinal designs, an advanced set of hydration assessment techniques (such as stable isotope dilution or neutron activation analysis) and more precise intake assessments to strengthen causal interpretations.

## 5 Conclusion

In conclusion, our study provides novel insights into the association between dietary creatine intake and hydration biomarkers in a large, nationally representative sample. The findings suggest a complex relationship between creatine consumption and systemic hydration, with both low and high creatine intake levels linked to altered plasma osmolality and hydration status. While moderate intake appears to have minimal effects on hydration markers, higher creatine intake is associated with reduced TBW, ICF, and ECF volumes. These results challenge the conventional notion that dietary creatine universally enhances body hydration and highlight the need for further research to clarify its role in fluid balance. Given the widespread use of creatine in nutrition and clinical settings, future studies should explore potential physiological mechanisms underlying these findings and assess the long‐term implications of dietary creatine on hydration homeostasis across different populations and health conditions.

## Author Contributions

**Sergej M. Ostojic:** conceptualization (equal), data curation (equal), formal analysis (equal), investigation (equal), methodology (equal), visualization (equal), writing – original draft (equal). **Erik Grasaas:** investigation (equal), methodology (equal), writing – review and editing (equal).

## Ethics Statement

Ethical approval for the NHANES study was granted by the National Center for Health Statistics Ethics Review Board under the following protocols: #98‐12, #2005‐06, Continuation of Protocol #2005‐06, #2011‐17, Continuation of Protocol #2011‐17, #2018‐01, Continuation of Protocol #2018‐01, and #2021‐05. Informed consent was obtained from all participants prior to their inclusion in the study. The research was conducted ethically following the World Medical Association Declaration of Helsinki.

## Conflicts of Interest

S.M.O. serves as a member of the Scientific Advisory Board on Creatine in Health and Medicine (AlzChem LLC). S.M.O. co‐owns patent “Supplements Based on Liquid Creatine” at the European Patent Office (WO2019150323 A1) and patent application “Composition Comprising Creatine for Use in Telomere Lengthening” at the US Patent and Trademark Office (# 63/608,850). S.M.O. has received research support related to creatine during the past 36 months from the Ministry of Science, Technological Development and Innovation; Provincial Secretariat for Higher Education and Scientific Research; AlzChem GmbH; Kaneka Nutrients; ThermoLife International, and Vireo System Inc. S.M.O. does not own stocks and shares in any organization. E.G. declares no conflicts of interest.

## Data Availability

The datasets backing the conclusions in this article are accessible in a publicly available repository as detailed below. The authors do not possess the data. The National Health and Nutrition Examination Survey data can be obtained from the National Center for Health Statistics at https://www.cdc.gov/nchs/nhanes/index. Further inquiries can be directed to the corresponding author.
